# Complex Networks Models and Spectral Decomposition in the Analysis of Swimming Athletes’ Performance at Olympic Games

**DOI:** 10.3389/fphys.2019.01134

**Published:** 2019-09-03

**Authors:** Vanessa Helena Pereira-Ferrero, Theodore Gyle Lewis, Luciane Graziele Pereira Ferrero, Leonardo Tomazeli Duarte

**Affiliations:** ^1^School of Applied Sciences, University of Campinas, Limeira, Brazil; ^2^Center for Homeland Defense and Security, Naval Postgraduate School, Monterey, CA, United States

**Keywords:** network physiology, complex networks, spectral decomposition, swimming, athletes’ performance evaluation

## Abstract

This study aims to present complex network models which analyze professional swimmers of 50-m freestyle Olympic competitions, comparing characteristics and variables that are considered performance determinants. This comparative research includes Olympic medalists’ versus non-medalists’ behavior. Using data from 40 athletes with a mean age, weight and height of 26 ± 2.9 years, 87 ± 5.59 kg, 193 ± 3.85 cm, respectively, at the Olympics of 2000, 2004, 2008, 2012, and 2016 (16-year interval), we built two types of complex networks (graphs) for each edition, using mathematical correlations, metrics and the spectral decomposition analysis. It is possible to show that complex metrics behave differently between medalists and non-medalists. The spectral radius (SR) proved to be an important form of evaluation since in all 5 editions it was higher among medalists (SR results: 3.75, 3.5, 3.39, 2.91, and 3.66) compared to non-medalists (2.18, 2.51, 2.23, 2.07, and 2.04), with significantly differences between. This study introduces a remarkable tool in the evaluation of the performance of groups of swimming athletes by complex networks, and is relevant to athletes, coaches, and even amateurs, regarding how individual variables relate to competition results and are reflected in the SR for the best performance. In addition, this is a general method and may, in the future, be developed in the analysis of other competitive sports.

## Introduction

Swimming is a kind of sport which involves agile body mechanics, including the action and reaction of Newton’s third law (Cureton, 1930) where the human body must combine physiology with engineering concepts to work as a whole (Alexander, 1992). Although it is one of the oldest physical activities, the first swimming competition (Porter, 2017) was only hosted in 1837 at London’s six artificial pools. The Olympic Era, in Athens, started in 1896, at a male-only swimming competition (Oppenheim, 1970). The Summer Olympic Games are a topic which has been attracting the attention of both general audiences and researchers. However, the Social Sciences have generated the most Olympic papers at 1,155 papers, while Exact Sciences like Engineering, have produced only 510 (Skibb et al., 2016). Considering the research involving swimming, until 2013 performance increased each year for both women and men in the World Championships and the Olympics (Wild et al., 2014), which points to the demand for evaluation of the parameters for understanding this growing performance behavior. Studies in this issue have made valuable contributions; however, there is still no consensus for the most knowledgeable way of studying the performance of athletes.

It is also important to consider that, regarding sport investigations, the multiple variable approach for the assessment of cardiorespiratory coordination and the effects on performance helps in the evaluation of different health and fitness training interventions (Garcia-Retortillo et al., 2019). In addition, there are interesting works in the literature that identified and created the first physiological complex models representing body changes and interactions among several organ systems. We can cite one of the first physiological networks created in the analysis of sleep stages, where each network node represented a type of body change interacting each other (Bashan et al., 2012). Some authors were also able to relate different aspects of physiological regulation using non-linear dynamics methods (Bartsch and Ivanov, 2014). The changes in brain, cardiac, and respiratory systems presented an important and strong relationship between network connectivity, links’ weights and physiologic functions. An interesting investigation about the dynamic interactions between organs developed the concept of Time Delay Stability, using complex hierarchical reorganization in network models (Bartsch et al., 2015). The authors proposed transitions across physiological states, and the network models represented various interactions, for example, between the brain and different organs. Statistical tools have also played an important role, once they were able to capture key elements throughout dynamic physiological interactions (Lin et al., 2016).

With the aim of contributing to the analysis of swimming performance, we here provide an investigation by considering the methodology of complex networks. In simple terms, these networks are mathematical graphs, which usually represent athletic performance parameters as nodes (vertices) and interactions as links (edges). Due to their flexibility, complex networks have been applied to a variety of scientific contexts, despite the fact that are still a lack of studies involving sports evaluation (Lewis, 2009). Considering complex social networks, the players were studied as nodes and the amount of interactions between them as links (Passos et al., 2011). Such investigation identified a higher number of interactions among efficient players. Another paper, inspired by the mentioned research on network physiology, investigated the fatigue occurrence during tethered running (Pereira et al., 2015). The network metrics assisted in the understanding of performance and in the avoiding of fatigue occurrence. Variables like Power, Velocity and Lactate time were highlighted. Additionally, considering sprinter athletes running in track field tests, the complex network models revealed that aerobic and anthropometric measures are meaningful in mathematical models and emphasized the extent of the comprehension of an entire complex context for an optimal performance output (Pereira et al., 2018). However, in the literature, the swimming topic through complex network analysis has not yet been explored in the context of the possibilities provided by such a remarkable approach.

Similar to the mentioned complex models, the network approach offers an overview of the athletes’ parameters during a competition scenario for swimming analysis. Shortly, a complex network model can be represented by a mathematical graph, which has an adjacency matrix. It is also possible to determine its decomposition system, for the spectral decomposition analysis. Through the matrix, the eigenvalue can be calculated for each network node, and the higher network eigenvalue is called Spectral Radius (SR). Through the groups of medalists versus non-medalists comparison, the spectral decomposition here proposed becomes possible to analyze the robustness of the interactions. All of this assists in performance comprehension. Physiological and related variables represented as nodes in the networks are one of the first steps in a distinguished level of swimming performance interpretation. If compared with isolated cause and effect studies, variables’ interactions are able to closely represent different levels of change during an activity or physical exercise.

In this article, inspired by the mentioned complex networks approaches on physiology and sports analysis, we developed a complex network study of the most recent 50 m freestyle swimming performance at the Olympic Games, Rio 2016, compared to London 2012, Beijing 2008, Athens 2004, and Sydney 2000. This 16 year range is useful to understand the medalists (winners) versus non-medalists’ behavior. Specifically, topological properties are used to summarize the impact of topology on behavior (Lewis, 2009), but not yet in sports like swimming in the Olympics, as introduced in this research.

## Materials and Methods

Considering swimming as a kind of exercise which depends on muscle contractility, strength, and speed through previously applied maximal or submaximal loads on the muscle system (Cuenca-Fernández et al., 2018) and metabolic responses (Hellard et al., 2018), data were selected from that which was publicly available from the last 5 editions of the games, specifically concerning the Men’s 50 m freestyle swimming, whose finalists are 8 athletes in each year, totalizing 40 athletes. Using data from swimmers with a mean age, weight and height of 26 ± 2.9 years, 87 ± 5.59 kg, 193 ± 3.85 cm, respectively, it was possible to organize the following data available for each athlete in the event: Birth year (YYYY), Age (years), Weight (kg), Height (cm), BMI – Body Mass Index (kg/m^2^), Number of Olympic Medals, Time (s), Velocity (m/s), Reaction Time (s) and Lane. Other available data from the country related to the athlete at the competition occasion included: Number of Women and Number of Men at the swimming team, Number of Total Participants, Number of Country Medals and Country HDI (Human Development Index).

Thereafter, it was necessary to proceed with the organization of data with the 8 finalists of each edition, leading to the correlations’ calculation and model construction. The 40 finalist athletes of the Men’s 50 m freestyle swimming competition, in a range of 16 years, were selected from the largest swimming competition that occurs every 4 years. These athletes were filtered through pre-Olympic qualifiers and then each staged within the specific event until the grand finale, where only 8 can compete, representing the swimming elite, with a small variance. The other filter of these athletes is that they should be specialized in high intensity and short duration exercise, since this test is crossing an Olympic pool of 50 m in about 20 s. It was also possible to work with current competition data from athletes representing the world’s greatest sprinter swimmers over 5 competitions, something that could be different from other groups of volunteers, usually from the same country, as that even though they were good swimmers, they would have a different kind of representativeness. It is believed, therefore, that the 2000, 2004, 2008, 2012, and 2016 Olympics finalists are high-level representatives of outstanding swimming performance.

The dataset comparisons via Pearson’s correlation were calculated. We firstly built two complex networks for each of the 5 editions of the games: groups of medalists and non-medalists dataset. The values of the correlations were the value of the network links in %. In order to set a complex model that adequately represents an analysis linked to individual performance, publicly available data were chosen. In this way, 6 variables were selected and transformed into nodes of the complex models. They are: Age (years), Weight (kg), Height (cm), BMI – Body Mass Index (kg/m^2^), Number of Olympic Medals and Reaction Time (s). The parameters which may directly predict victory, such as Velocity and Time, were not transformed into nodes, in order to avoid bias results. The selection of the variables was made considering those related to performance and individualizing each athlete, according to the Data Availability of each competition. Variables considered unrelated to performance were not included, such as team size and lane. In addition, there is great importance identified in runners for anthropometric data (Pereira et al., 2018). In accordance with the concept of creating complex networks, it is of fundamental importance to include variables such as height, weight, age, BMI, etc. The idea is to understand the relationships (information flow) among them and other variables. Such relationships are represented by links and the variables under analysis represented by nodes, which result in the final complex structure under analysis.

Five correlations (5 links) were calculated for each of the 6 variable analyzed (6 nodes) with a total of 30 links by each of 10 network model. The total calculation involves the creation of: 10 complex networks, 60 nodes, and 300 weighted links (correlations).

Any network can be represented by a graph. Any graph can be represented by its adjacency matrix, from which other matrices such as Laplacian are derived. This linear algebra determines that for each matrix, a collection of eigenvalues with their respective eigenvectors can be associated. The term Eigen has a German origin and means what is inherent, a characteristic or fundamental property. Therefore, knowing that each graph is represented by its matrix, it is natural to investigate its “Eigen system” once it characterizes the graph (Van Mieghem, 2014). Other topological graph characteristics are used to characterize network connectivity, for example, in financial market fluctuations (Spelta, 2017). Topological metrics can be classified into metrics that are based on graph distance, connectivity and spectrum (Van Mieghem, 2010; Jovanovic et al., 2017). The nature of a complex system pattern is possible to determine by decomposing the system’s response to a stimulus into a set of fundamental modes or basis vectors also called orthonormal vectors. This mathematical process is called spectral decomposition. This process of finding the basic vibrational modes (harmonics) and expressing them in terms of constants is called spectral analysis.

This kind of complex network analysis refers to the analysis of a mathematical graph. The measure of the degree of the nodes (parameters under analysis) of a complex network (graph) is related to the total number of edges (relations between the nodes) incident to this node. Nodes with an higher number of edges to it incidents are called hubs. Only the measure of nodes’ degree may not adequately reflect the importance of these nodes in the complex model. An alternative metric can be used to calculate the eigenvalues for each node of the resulting network and to rank these eigenvalues for the General Winners network. Each metric, such as eigenvalue calculation, was made by Eclipse IDE via Java Programing algorithm. The eigenvalue calculation is crucial for the understanding of this approach, and it is explained at the introductory section. The largest eigenvalue of a graph is also known as its SR or index. The basic information about the largest eigenvalue of a (possibly directed) graph is provided by Perron–Frobenius theory (Smyth, 2002).

Each graph *G* has a real eigenvalue θ_0_ with non-negative real corresponding eigenvector, and such that for each eigenvalue θ we have |θ| ≤ θ_0_. The value θ_0_ (*G*) does not increase when vertices or edges are removed from *G* (Brouwer and Haemers, 2011).

Under the assumption that *G* is strongly connected, then:

(i) θ_0_ has multiplicity 1.
(ii) If *G* is primitive (strongly connected, and such that not all cycles have a length that is a multiple of some integer *d* > 1), then | θ | < θ_0_ for all eigenvalues θ different from θ_0_.
(iii) The value θ_0_ (*G*) decreases when vertices or edges are removed from *G*.

There are essentially two types of information related to the spectrum. The largest eigenvalues (and their eigenspaces) give some information on global graph properties. The typical eigenvalues give information on local graph properties, such as degree, partition function, etc. Here the focus is on the SR, which is related to a graph property called maximum eigenvalue. The maximum eigenvalue of a complex network (graph) is also called the SR. As mentioned in the explanation of its calculation, the eigenvalue is a final value assigned to each node, considering not only the number of edges (links) of this node but also the weight of the links to it, associated to its location in the complex network topology. A node with final high value of eigenvalue, if compared to the others in the same graph, is interpreted as an important node due to the amount and weight of the edges, besides it being a node that connects to the nodes around it that, in turn, also have greater amount of weighted links and so on, relevance. In this new proposal of spectral decomposition, considering that the correlation matrix in the context of the networks is sensitive to the weight value attributed to the link, we used correlations in an attempt to not discard connections which have their importance in the context of the complex model and the calculation of the SR. The idea was to consider the set of interactions and their outcomes. That is the reason why complex network analysis includes such measure and the greater adequacy of a measure of maximum eigenvalue (SR) to a convergence tendency of the complex system as a whole. The data that support the analysis and conclusions of this article are publicly available on the website Sports Reference (Evans et al., 2016) and are in accordance with all the Publishing Ethics of this journal.

## Results

The following public data available at every edition of the games was analyzed. The measures of central tendency by each Olympic game edition are shown at the Table 1.

**TABLE 1:** Measures of central tendency – mean and standard deviation – by each Olympic edition split by medalists and non-medalists athletes.

					**Weight**	**Height**	**BMI**	**Olympic**	**Reaction**		**Velocity**
**Olympiad**	**Year**	**DataSet**	**MCT^∗^**	**Age**	**(kg)**	**(cm)**	**(kg/m^2^)**	**medals**	**time**	**Time (s)**	**(m/s)**
Sydney	2000	Medalists	Mean	22.000	86.333	194.000	22.886	7.000	0.743	21.997	2.273
			SD	2.000	8.444	2.667	1.577	2.000	0.051	0.022	0.002
		Non-med	Mean	26.200	88.200	196.800	22.802	1.800	0.824	22.298	2.242
			SD	2.240	2.640	3.520	1.042	2.880	0.033	0.130	0.013
Athens	2004	Medalists	Mean	24.667	89.667	195.000	23.560	4.667	0.673	21.963	2.277
			SD	2.889	6.222	3.333	1.128	3.556	0.036	0.038	0.004
		Non-med	Mean	27.400	88.400	192.000	24.027	1.600	0.704	22.200	2.252
			SD	2.160	3.840	4.000	1.594	2.560	0.053	0.092	0.009
Beijing	2008	Medalists	Mean	22.667	86.000	197.667	22.011	3.667	0.720	21.413	2.335
			SD	1.556	4.000	2.889	0.944	0.444	0.027	0.076	0.008
		Non-med	Mean	25.600	84.600	190.600	23.281	1.800	0.686	21.660	2.308
			SD	2.480	2.880	1.920	0.767	1.040	0.042	0.028	0.003
London	2012	Medalists	Mean	24.667	91.333	196.333	23.674	2.667	0.637	21.490	2.327
			SD	2.444	7.556	1.778	1.757	1.111	0.016	0.100	0.011
		Non-med	Mean	28.200	78.600	190.600	21.670	1.800	0.690	21.798	2.294
			SD	2.960	2.720	2.320	1.188	1.040	0.040	0.082	0.009
Rio	2016	Medalists	Mean	29.000	92.667	196.000	24.041	4.333	0.670	21.433	2.333
			SD	4.000	10.444	3.333	1.956	2.444	0.027	0.038	0.004
		Non-med	Mean	23.800	86.200	190.600	23.745	0.000	0.648	21.816	2.292
			SD	1.840	3.440	2.320	1.167	0.000	0.046	0.106	0.011

*^∗^Measures of central tendency – mean and standard deviation – by each Olympic edition, split by medalists and non-medalists athletes.*

**FIGURE 1:**
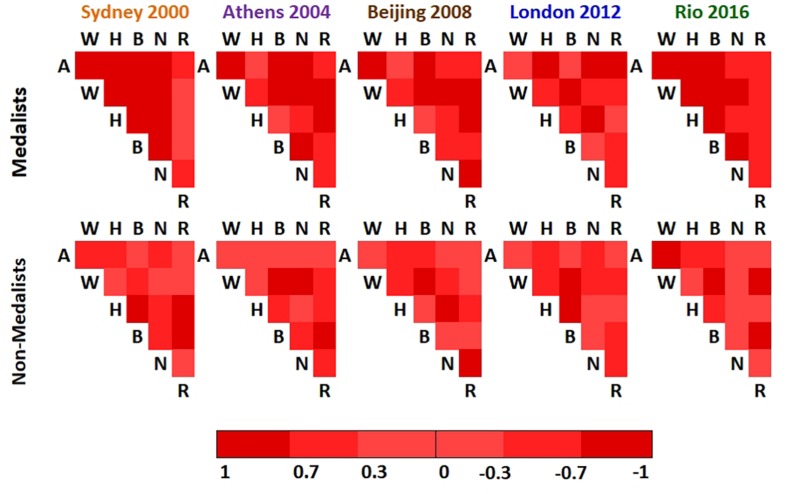
The heat map of the Pearson’s correlations results among variables for each edition and complex network model. Predominantly, the weighted links between Medalists had highest values than the weighted links of non-medalists athletes, with significant differences between groups (One-way ANOVA followed by Tukey HSD *post hoc* test, *p* < 0.01 at 2000, 2004, and 2008 editions and *p* < 0.05 at 2012 and 2016 editions). In this figure, A, age; W, weight (kg); H, height (cm); B, body mass index (kg/m^2^); N, number of Olympic medals and R, reaction time (s).

Pearson’s correlations among sets of data were defined as the weights of the network. Such correlations were a useful choice once it showed the same result for both directions of the model set, as shown at the Figure 1.

**FIGURE 2:**
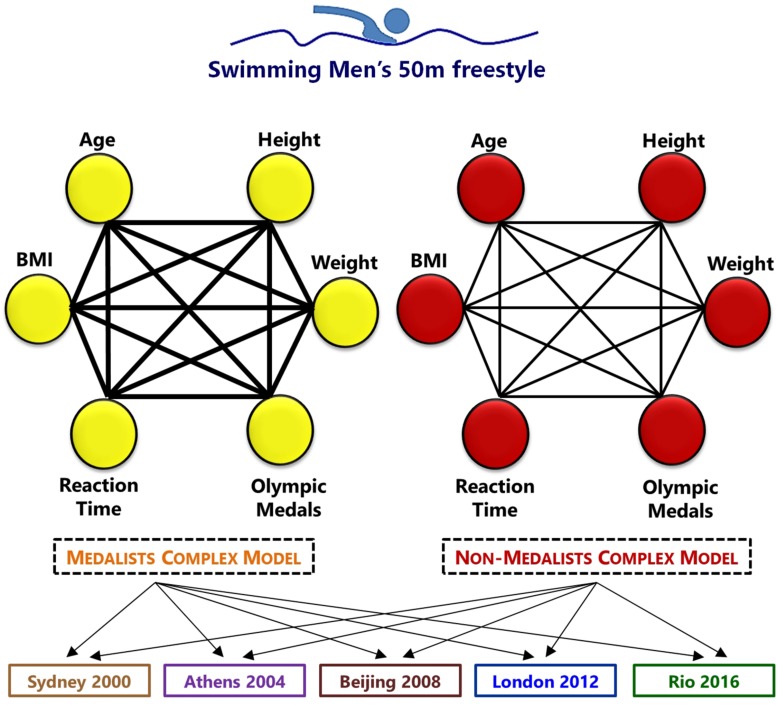
Complex network model parameters (nodes and links) representation proposed for Medalists (yellow) and non-medalists (red) athletes. Every edition of the games results in two complex models. They are: The 2000 Olympic Games, which took place at Australia, in Sydney; 2004 Olympic Games, which took place in Greece, at Athens city; the 2008 Olympic Games, which took place in China, in Beijing; the 2012 Olympic Games, which took place in United Kingdom, in London; and the 2016 Olympic Games, which took place in Brazil, in the city of Rio de Janeiro. The finalists of the 50 m freestyle male swimmers were considered, relating 6 transformed variables into nodes – colored (Age, Height, Weight, BMI, Number of Olympic Medals and Reaction Time) and their weighted links (black) through the resulting correlation. The links of the Medalists complex models had higher weighted links.

A similar method was used in another research of the authors (Pereira et al., 2015). An algorithm was built in Java Programing Language, which received the data sets as vectors in a main function, passing them to the function to correlation calculation including the set of data gathered. Every value of correlation was considered and transformed into link. For example, if the result of the correlation between Age and BMI was 0.56, a connection (link) was added between such nodes with a weight of 56%. The 6 nodes of each Olympic edition were included with the same magnitude, once one of the main goals of the model is to identify the resulting dynamics of the nodes through complex metrics. The resulting weighted network had bidirectional links, which means that the link influence flows in both directions. This was necessary once it is not possible to stand that a node like Age, for example, has a cause and effect weight in BMI. Instead, it has a correlation inside the dynamic network. It is interesting to note that most real-world networks, links’ weights may mean capacity, flow or intensity (Bartsch et al., 2015). In this way, a complex model is analyzable simplification of reality representation with mathematical groundwork. Such links’ weights in every complex model directly determined the network structure and the main complex metric utilized in the result: The SR. The complex network models were built as shown in Figure 2.

With the focus on what data to analyze together, from the point of view of complex network construction, and the spectral decomposition, which can help to identify trends in the profile of the winners, the complex networks were built in a computational interface. Then, the network SRs were determined. The SR is computed by finding the largest eigenvalue of the weighted connection matrix C, where an element of C is equal to the weight assigned to the link between nodes. Matrix C is symmetric, because links are bi-directional.

By considering the 6 nodes and 10 models proposition, 2 networks by edition were constructed – 2 networks for each edition (2 networks for Sydney, 2 networks for Athens, 2 networks for Beijing, 2 networks for London and 2 networks for Rio). The core idea was to compare medalists’ and non-medalists’ behavior via complex models. The Winners – Medalists network has the correlations of the 3 medalists for that edition. The non-medalists network was created by data from the 5 other athletes’ positions, which did not win medals.

**FIGURE 3:**
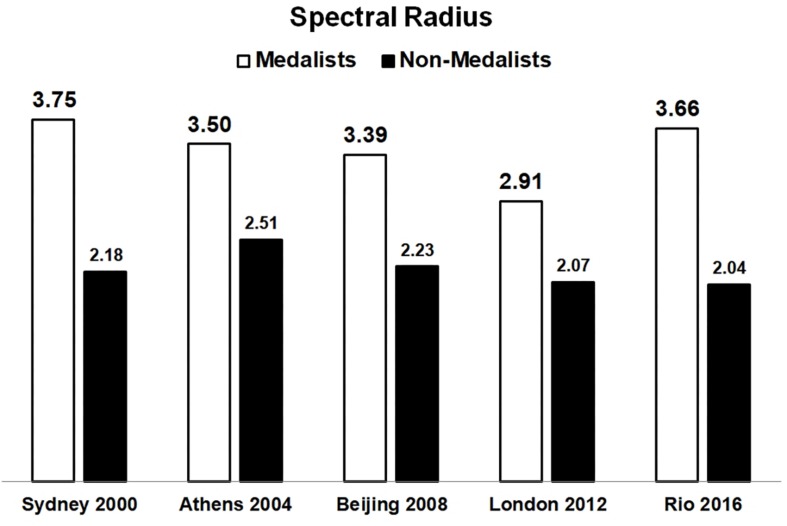
The Spectral Radius (SR) network (*y*-axis) by Olympic edition, comparing Medalists result (white) and non-medalists result (black). The Medalists – Winners network of each edition had the highest SR values at all competitions. It is worth mentioning that the winners always resulted in higher SR values at all editions analyzed, with significantly differences between groups (One-way ANOVA followed by Tukey HSD *post hoc* test, *p* < 0.05).

**FIGURE 4:**
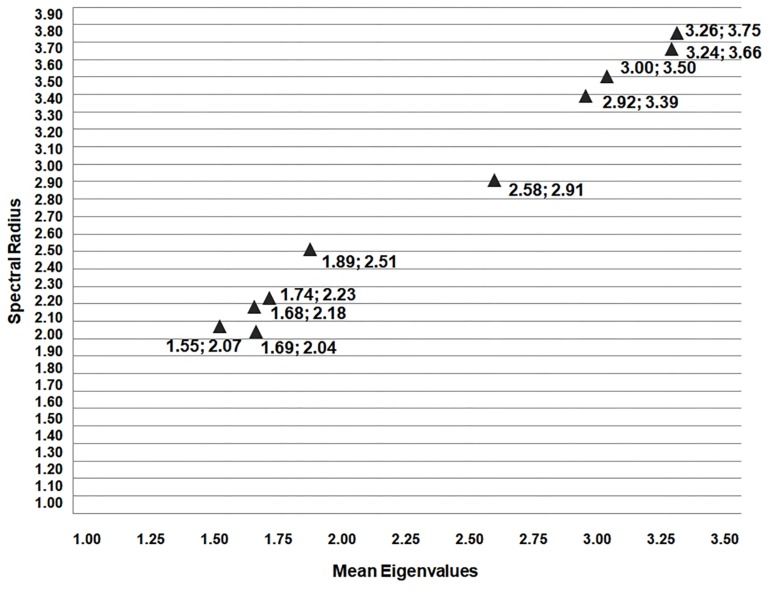
The SR results (*y*-axis) by Olympic edition versus mean eigenvalues (*x*-axis) represented by the triangle with the coordinates (*x*; *y*). It is important to note that the Medalists SR results were always above 2.91 and the non-medalists’ results are under 2.51. This point to a common behavior among Medalists-Winners (highest values) at all 5 editions of the games analyzed, with significant differences (One-way ANOVA followed by Tukey HSD *post hoc* test, *p* < 0.05).

For comparative analysis, within these new 10 complex models we found the eigenvalues of each node and the value of the SR (largest eigenvalue) for each network. In fact, the SR of the winners’ networks, in each edition, are predominantly bigger than the SR of the non-medalists’ networks, as shown at Figures 3, 4. The SR proved to be an important form of evaluation since in all 5 editions it was higher among medalists (SR results: 3.75; 3.5; 3.39; 2.91; and 3.66) compared to non-medalists (2.18; 2.51; 2.23; 2.07; and 2.04), with significant differences between groups (One-way ANOVA followed by Tukey HSD *post hoc* test, *p* < 0.05).

## Discussion

Through this new study using quantitative data for the construction of complex networks models, it is interesting to note that success and winning in sports are reached by decisions guided by data and models. Sports analytics is a process of strategically modeling the data available to transform it into a source of competitive advantage (Trewin et al., 2004; Miller, 2015). Players, managers, owners and fans are interested in such strategies in the context of data science, where sports analytics is a blend of business savvy, information technology and modeling techniques.

Our network approach moves toward the recent understanding of the human body as a collection of physiologically interacting systems, according to the interdisciplinary concept of network physiology (Ivanov et al., 2016). Networks are representations of these interacting physiological systems, which include organ changes and metabolites, among others, and provide feedback and feedforward data interacting, which are capable of reflecting on different performances. The computational modeling comes to help in this understanding, allowing the calculation of complex metrics like SR, for example. The concept of network physiology in different sports is still not fully explored but is promising, and may in the future also involve specific interactions, such as those found in different brain regions and their effects on physiological states during sleep (Liu et al., 2015). Considering the potential limitations of this work, there is the fact that the data were not obtained in time series and successively, as could be done in a hypothetical scenario of competition. On the other hand, it was possible to work with actual competition data for athletes representing the world swimming elite over 5 distinct competitions (16 years range) from different countries.

This research approach brings a novel way in order to identifying data sources, gathering data to organize and prepare for the complex analysis. Furthermore, the data selections for this study seemed to be in concordance with research involving the need of evaluate anthropometrics and athletes’ variables. For example, evaluating the performance and the anthropometrical parameters, such as body height, called the attention for both female swimmers (Jagomägi and Jürimäe, 2005) and male swimmers 100-m events (Sammoud et al., 2018). In addition, age, height, and hand grip strength were the best predictors in short-distance events (Zampagni et al., 2008). A balanced diet allows to maintain a stable body weight for athletic performance in swimmers (Ciosek et al., 2015) and anaerobic qualities are important in regards to age in other competitions (Fairbrother, 2007). These are shown at the Olympics over the years, with the important contribution to the model body demonstrated as interactions of a complex network (Herman et al., 2009; Ivanov et al., 2016).

The complex networks make possible the study of the dynamical interactions among professional athletes. This article took into account 5 different Olympics editions and allowed the calculation of the SR, which is a measure that reflects the robustness of each complex model. Once multiple seasons were analyzed, it is possible to track the development of winners SR values and its similarities. In the case of the models of the medalists, there is a communication of greater weight among the variables, that is, for a winner; the intensity of communication between variables at the proposed levels was reflected in a higher SR. For non-medalists, this lower level of communication among the variables may have been decisive in the position they reached.

By considering the SR values analysis, the winners’ networks always have the highest SR values. It is also true even when considering the mean eigenvalues. Winners may be better at combining all factors, here represented as nodes. Maybe a well-balanced athlete is a winner and the complex networks and SR analysis are a newsworthy way of identification of the best fit athlete. It is interesting to note that the variables analyzed via complex models together, may indicate the best use of the set of factors by the winners. Thus, complex networks in association with complex metrics, such as SR, may, in the future, allow for a given test, according to the specific profile and performance of the analyzed athletes, the calculation of SR for different groups of athletes in training and determination of SR values. These network models and their metrics can assist in verifying which groups of athletes would present a greater chance of victory when compared to each other.

The higher SR value among medalists should reflect the more efficient communication of the variables analyzed within the model. The combined communication between physiological basis and previous experience, results in a higher SR for medalists. The application of these physiological complex network models should be taken into consideration when focusing on new training strategies, assisting coaches, athletes, and amateurs. The complex models in conjunction with the spectral analysis proposed by this study showed consistency with the profile of the winners. Such analysis can be applied in future work for women’s swimming events and also for other sports categories, such as athletics. The methodology presented here can also be applied in other types of tests and even other sports, in order to identify the profiles of possible medalist groups and may help in practice, which may inspire new future research applications.

## Data Availability

All datasets generated for this study are included in the manuscript and/or the supplementary files.

## Ethics Statement

Ethical review and approval was not required for the study on human participants in accordance with the local legislation and institutional requirements. Written informed consent for participation was not required for this study in accordance with the national legislation and the institutional requirements.

## Author Contributions

VP-F, TL, LF, and LD proposed models ideas, interpreted the data, and created the models. VP-F, LF, and LD wrote the main manuscript text and prepared the figures. TL and VP-F developed the network software, designed and built complex models, and made metrics. All authors reviewed the manuscript.

## Conflict of Interest Statement

The authors declare that the research was conducted in the absence of any commercial or financial relationships that could be construed as a potential conflict of interest.

## References

[B1] Alexander R. M. (1992). *The Human Machine: How the Body Works.* Columbia: Columbia University Press.

[B2] Bartsch R. P. Ivanov P. C. (2014). “Coexisting forms of coupling and phase-transitions in physiological networks,” in *International Conference on Nonlinear Dynamics of Electronic Systems*, eds Mladenov V. M. Ivanov P. C., (Berlin: Springer), 270–287. 10.1007/978-3-319-08672-9-33

[B3] Bartsch R. P. Liu K. K. Bashan A. Ivanov P. C. H. (2015). Network physiology: how organ systems dynamically interact. *PLoS One* 10:e0142143. 10.1371/journal.pone.0142143 26555073 PMC4640580

[B4] Bashan A. Bartsch R. P. Kantelhardt J. W. Havlin S. Ivanov P. C. H. (2012). Network physiology reveals relations between network topology and physiological function. *Nat. Commun.* 3:702. 10.1038/ncomms1705 22426223 PMC3518900

[B5] Brouwer A. E. Haemers W. H. (2011). *Spectra of Graphs.* Berlin: Springer Science & Business Media.

[B6] Ciosek Z. Drozd A. Lubkowska A. (2015). Dynamics of changes body composition of polish national swimming team during the one month of training camp prior the junior world championship in Dubai in 2013. *Pomeranian J. Life Sci.* 61 232–236. 27141612

[B7] Cuenca-Fernández F. Ruiz-Teba A. López-Contreras G. Arellano R. (2018). Effects of 2 types of activation protocols based on postactivation potentiation on 50-m freestyle performance. *J. Strength Cond. Res.* 10.1519/JSC.0000000000002698 [Epub ahead of print]. 33105381

[B8] Cureton T. K. Jr., (1930). Mechanics and kinesiology of swimming: the crawl flutter kick. *Res. Q. Am. Phys. Educ. Assoc.* 1 87–121. 10.1080/23267402.1930.10625804

[B9] Evans H. Gjerde A. Heijmans J. Mallon B. (2016). *Sports Reference – Olympic Sports Database 2016.* Available at: https://www.sports-reference.com/olympics (accessed November 1, 2018).

[B10] Fairbrother J. T. (2007). Age-related changes in top-ten men’s US masters 50-m freestyle swim times as a function of finishing place. *Percept. Mot. Skills* 105(Suppl. 3), 1289–1293. 10.2466/pms.105.4.1289-1293 18380129

[B11] Garcia-Retortillo S. Gacto M. O’Leary T. J. Noon M. Hristovski R. Balagué N. (2019). Cardiorespiratory coordination reveals training-specific physiological adaptations. *Eur. J. Appl. Physiol.* 119 1701–1709. 10.1007/s00421-019-04160-4163 31187282

[B12] Hellard P. Pla R. Rodríguez F. A. Simbana D. Pyne D. B. (2018). Dynamics of the metabolic response during a competitive 100-m freestyle in elite male swimmers. *Int. J. Sports. Physiol. Perform.* 13 1011–1020. 10.1123/ijspp.2017-2597 29466071

[B13] Herman P. Kocsis L. Eke A. (2009). “Fractal characterization of complexity in dynamic signals: application to cerebral hemodynamics,” in *Dynamic Brain Imaging*, ed. Hyder F., (Totowa, NJ: Humana Press), 10.1007/978-1-59745-543-5-2 18839086

[B14] Ivanov P. C. Liu K. K. L. Bartsch R. P. (2016). Focus on the emerging new fields of network physiology and network medicine. *New J. Phys.* 18:100201. 10.1088/1367-2630/18/10/100201 30881198 PMC6415921

[B15] Jagomägi G. Jürimäe T. (2005). The influence of anthropometrical and flexibility parameters on the results of breaststroke swimming. *Anthropol. Anz.* 63 213–219. 10.1127/anthranz/63/2005/213 15962572

[B16] Jovanovic N. Jovanovic Z. Jevremovic A. (2017). “Complex networks analysis by spectral graph theory,” in *Proceedings of the Sinteza 2017 - International Scientific Conference on Information Technology and Data Related Research* (Belgrade: Singidunum University).

[B17] Lewis T. G. (2009). *Network Science: Theory and Applications.* Hoboken, NJ: John Wiley & Sons.

[B18] Lin A. Liu K. K. Bartsch R. P. Ivanov P. C. H. (2016). Delay-correlation landscape reveals characteristic time delays of brain rhythms and heart interactions. *Philos. Trans. A Math. Phys. Eng. Sci.* 374:2067. 10.1098/rsta.2015.0182 27044991 PMC4822443

[B19] Liu K. K. Bartsch R. P. Lin A. Mantegna R. N. Ivanov P. C. H. (2015). Plasticity of brain wave network interactions and evolution across physiologic states. *Front. Neural Circuits* 9:62. 10.3389/fncir.2015.00062 26578891 PMC4620446

[B20] Miller T. W. (2015). *Sports Analytics and Data Science: Winning the Game with Methods and Models.* Upper Saddle River, NJ: FT Press.

[B21] Oppenheim F. (1970). *The History of Swimming.* North Hollywood, CA: Swimming World.

[B22] Passos P. Davids K. Araújo D. Paz N. Minguéns J. Mendes J. (2011). Networks as a novel tool for studying team ball sports as complex socials systems. *J. Sci. Med. Sport* 14 170–176. 10.1016/j.jsams.2010.10.459 21145787

[B23] Pereira V. H. Gama M. C. Sousa F. A. Lewis T. G. Gobatto C. A. Manchado-Gobatto F. B. (2015). Complex network models reveal correlations among network metrics, exercise intensity and role of body changes in the fatigue process. *Sci. Rep.* 5:10489. 10.1038/srep10489 25994386 PMC4440209

[B24] Pereira V. H. Gobatto C. A. Lewis T. G. Ribeiro L. F. P. Beck W. R. Dos Reis I. G. M. (2018). Computational and complex network modeling for analysis of sprinter athletes’ performance in track field tests. *Front. Physiol.* 9:843. 10.3389/fphys.2018.00843 30034346 PMC6043640

[B25] Porter L. (2017). *The History of Competitive Swimming. Live Strong.* Available at: https://www.livestrong.com/article/342427-the-history-of-competitive-swimming/ (accessed May 12, 2019).

[B26] Sammoud S. Nevill A. M. Negra Y. Bouguezzi R. Chaabene H. Hachana Y. (2018). 100-m Breaststroke swimming performance in youth swimmers: the predictive value of anthropometrics. *Pediatr. Exerc. Sci.* 30 393–401. 10.1123/pes.2017-2220 29546801

[B27] Skibb R. Cressey D. Van Noorden R. (2016). Scholarly olympics: how the games have shaped research. *Nature* 536 18–20. 10.1038/536018a 27488783

[B28] Smyth M. R. F. (2002). A spectral theoretic proof of perron-frobenius. *Math. Proc. R. Ir. Acad.* 102 29–36.

[B29] Spelta A. (2017). Financial market predictability with tensor decomposition and links forecast. *Appl. Netw. Sci.* 2:7. 10.1007/s41109-017-0028-21 30443562 PMC6214239

[B30] Trewin C. B. Hopkins W. G. Pyne D. B. (2004). Relationship between world-ranking and olympic performance of swimmers. *J. Sports Sci.* 22 339–345. 10.1080/02640410310001641610 15161107

[B31] Van Mieghem P. (2010). *Graph Spectra for Complex Networks.* Cambridge: Cambridge University Press.

[B32] Van Mieghem P. (2014). *Performance Analysis of Complex Networks and Systems.* Cambridge: Cambridge University Press.

[B33] Wild S. Rüst C. A. Rosemann T. Knechtle B. (2014). Changes in sex difference in swimming speed in finalists at FINA world championships and the olympic games from 1992 to 2013. *BMC Sports Sci. Med. Rehabil.* 6:25. 10.1186/2052-1847-6-25 25120914 PMC4129435

[B34] Zampagni M. L. Casino D. Benelli P. Visani A. Marcacci M. De Vito G. (2008). Anthropometric and strength variables to predict freestyle performance times in elite master swimmers. *J. Strength Cond. Re.* 22 1298–1307. 10.1519/JSC.0b013e31816a597b 18545175

